# Reactivation of Hepatitis B Virus in HBsAg-Negative Patients with Hepatocellular Carcinoma

**DOI:** 10.1371/journal.pone.0122041

**Published:** 2015-04-20

**Authors:** Jeong Won Jang, Young Woon Kim, Sung Won Lee, Jung Hyun Kwon, Soon Woo Nam, Si Hyun Bae, Jong Young Choi, Seung Kew Yoon, Kyu Won Chung

**Affiliations:** 1 Department of Internal Medicine, Seoul St. Mary's Hospital, College of Medicine, The Catholic University of Korea, Seoul, Korea; 2 Department of Internal Medicine, The Catholic University of Korea Incheon St. Mary’s Hospital, Incheon, Korea; CRCL-INSERM, FRANCE

## Abstract

**Background & Aims:**

Despite increasing attention to hepatitis B virus (HBV) reactivation in hematologic settings, information on reactivation in hepatitis B surface (HBsAg)-negative patients with hepatocellular carcinoma (HCC) remains unknown. This study aimed to determine the incidence and risk factors of HBV reactivation in HBsAg-negative patients undergoing transarterial chemoembolization (TACE).

**Methods:**

A total of 109 HBsAg-negative patients with HCC were consecutively recruited for this study and treated with either mono- (n = 75), combination-drug TACE (n = 20), or combination-drug TACE plus radiotherapy (n = 14). With serial monitoring of virological markers every 2–3 months, patients were observed for HBV reactivation (defined as the reappearance of HBV DNA or sero-reversion of HBsAg) in comparison with control subjects with HBsAg-negative cirrhosis (n = 16) or HBsAg loss (n = 46).

**Results:**

During the study period, HBV reactivation occurred in 12 (11.0%) and 1 (1.6%) patients in the TACE and control groups, respectively. The median level of HBV DNA at reactivation was 5,174 copies/ml (range: 216–116,058). Of the 12 patients with HBV reactivation, four (33.3%) developed clinical hepatitis, including one patient who suffered from decompensation. All antiviral-treated patients achieved undetectable HBV DNA or HBsAg loss after commencement of antiviral drugs. TACE was significantly correlated with a high incidence of HBV reactivation, with increasing risk of reactivation with intensive treatment. On multivariate analysis, treatment intensity and a prior history of chronic hepatitis B remained independently predictive of reactivation.

**Conclusions:**

TACE can reactivate HBV replication in HBsAg-negative patients, with a dose-risk relationship between treatment intensity and reactivation. Patients with prior chronic HBV infection who are to undergo intensive TACE should be closely monitored, with an alternative approach of antiviral prophylaxis against HBV reactivation.

## Introduction

Hepatitis B virus (HBV) reactivation is a well-known, potentially life-threatening complication that is often encountered in chronic hepatitis B surface antigen (HBsAg) carriers receiving cancer chemotherapy. The impaired immunity associated with either the underlying malignancy or chemotherapy is a key factor that predisposes these patients to the development of viral reactivations. Although this phenomenon has been primarily observed in hematological diseases [1], its clinical spectrum has greatly expanded as a result of newer, potent immunosuppressive therapies for varying diseases. Data from a pooled analysis of hepatocellular carcinoma (HCC) patients have shown a rate of reactivation ranging from 4.3 to 67% [1,2], indicating the relatively high risk of reactivation in HBsAg-positive HCC patients.

Given the observation that the frequency and severity of HBV reactivation are enhanced by the degree of immunosuppression caused by therapy [1,3], host immunity severely down-regulated under certain intensive therapies could potentially lead to HBV reactivation even in HBsAg-negative patients with a prior resolved HBV infection. Such cases of de novo hepatitis B reactivation have often been documented in HBsAg-negative individuals undergoing rituximab-containing chemotherapy or hemato-poietic stem cell transplantation [4–6].

The development of HBV reactivation in the setting of HCC is linked directly to poor HCC survival [7]. Furthermore, hepatic morbidity due to reactivation results in treatment disruptions of transarterial chemoembolization (TACE) [8], potentially adversely affecting patient outcome. Since HCC frequently arises from a background of cirrhosis, enhanced viral replication in conjunction with TACE-induced liver damage can have an additive effect on the liver inflammatory microenvironment, thereby facilitating tumor progression on TACE [9]. Collectively, the overall findings warrant special attention to patients with HCC as high-risk situations. It is unfortunate that, despite the multitude of reports showing the relatively high risk of reactivation under anti-HCC treatments [2], there are no specific recommendations in practice guidelines for reactivated diseases during TACE, which is the mainstay treatment for unresectable HCC.

Pre-emptive antiviral therapy before chemotherapy is currently recommended to reduce HBV reactivation in HBsAg-positive patients [10–12]. Nevertheless, this recommendation is debated in HBsAg-negative patients on chemotherapy, largely due to lack of confirmative data. To date, information on HBV reactivation in HBsAg-negative patients with HCC has been limited to case reports or small case series [13,14]. No study has yet to define high-risk patients, or preventive measures for HBV reactivation in these settings. Therefore, this study was conducted to provide detailed information on the frequency and clinical manifestation of HBV reactivation in HBsAg-negative patients who undergo TACE. With the analysis of risk factors, we also identified high-risk patients for preventive measures against HBV reactivation.

## Patients and Methods

### Patients

This was a retrospective analysis of prospectively collected dataset on HBsAg-negative patients diagnosed with HCC who underwent transarterial chemotherapy at our liver units from July 2006 to December 2012. As a treatment for unresectable HCC, TACE was selected mainly for patients with multifocal tumors. Patients with portal vein thrombosis (PVT) or extrahepatic metastasis were considered to receive radiotherapy (three-dimensional conformal radiotherapy or helical tomotherapy) or systemic chemotherapy in addition to TACE [15].

Patients who had any of the following criteria were excluded from the study: positive HBsAg or detectable serum HBV DNA level before treatment; co-existing serious medical diseases; positive tests for human immunodeficiency virus; altered blood cell counts (white blood cell < 3,000/mm^3^ or platelet count < 50,000/mm^3^); or pre-existing evidence of hepatic decompensation including encephalopathy, prolonged prothrombin time (> 3 s), or a bilirubin level > 2.5 times the upper limit of normal (ULN). Each patient provided written informed consent for this study. The study was approved by the Ethics Committee of the Catholic University of Korea.

### Treatment and follow-up

The intensity of TACE protocols used to treat HCC was determined based on tumor stage, as described elsewhere [15]: intra-arterial doxorubicin (50 mg) mono-therapy for multifocal tumors with/without peripheral PVT (mono-TACE); combination-drug chemotherapy using intra-arterial epirubicin (50 mg) and cisplatin (60 mg) plus systemic 5-fluorouracil (200 mg) infusion for large tumors (> 10 cm) or PVT at the first/second branch (combo-TACE); combo-TACE plus radiotherapy for a large PVT and/or extrahepatic metastasis (combo + RT). The transarterial procedure was repeated until complete necrosis of the viable tumor was achieved.

Serum HBsAg was measured using the ARCHITECT quantitative assay (detection range: 0.05–250 IU / mL; Abbott Park, IL, USA). Hepatitis B e antigen (HBeAg), anti-HBe, and anti-HBs were tested by commercial immunoassays (Abbott Laboratories, Abbott Park, IL). Serum HBV DNA was quantified by COBAS Ampli-Prep-COBAS TaqMan (detection limit: 116 copies/ml; Roche Diagnostic, Branchburg, NJ). Serologic markers and HBV DNA were monitored every 2 to 3 months up to 2 years after study enrollment and whenever clinically warranted for patients, as described below.

HBV reactivation was defined as the appearance of serum HBV DNA or HBsAg sero-reversion. Hepatitis due to HBV reactivation was defined as a ≥ 3-fold increase in ALT levels. To exclude treatment-related hepatitis, an abrupt elevation in ALT levels within 2 weeks after treatment was not considered to indicate reactivation hepatitis. During the study period, the Korean national health insurance system only covered viremic carriers (HBV DNA > 10^4–5^ copies/ml) with increased aminotransferase levels (> 2 × ULN). Thus, patients with reactivation were administered antiviral drugs upon fulfillment of these criteria. Additionally, for patients who did not meet the insurance criteria, antiviral therapy was initiated for patients who agreed to have treatment without reimbursement.

### Statistical analysis

The duration of follow-up was calculated from the initiation of treatment to the onset of reactivation, death, or the last visit up to 2 years. The cumulative event of HBV reactivation was estimated using the Kaplan—Meier method, and the differences were analyzed using the log-rank test. Univariate and multivariate analyses with the Cox proportional hazard model were used to identify risk factors for HBV reactivation. Two-tailed P values less than 0.05 were considered significant. Data were analyzed using the SPSS 15.0.

## Results

### Study population

**Fig 1:**
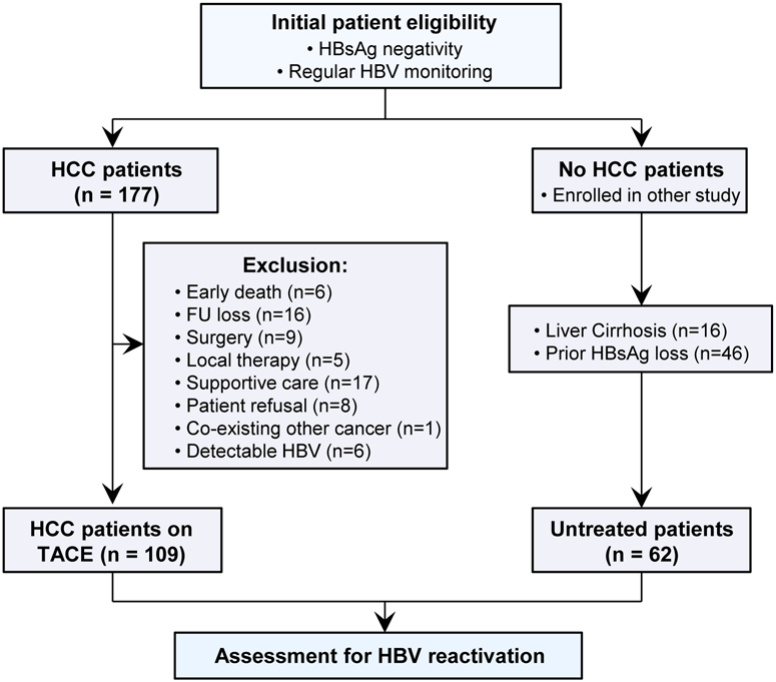
Patient enrollment and assessment.

A total of 177 HBsAg-negative patients with HCC were consecutively evaluated for study enrollment. Among them, 68 patients were excluded from the analysis due to early death within 3 months (6 patients), transfer or loss to follow-up (16 patients), surgery following TACE (9 patients), local ablative therapy (5 patients), supportive care (17 patients), patient refusal (8 patients), co-existing other malignancy (1 patient), or detectable HBV DNA levels (6 patients) at diagnosis of HCC. According to the aforementioned treatment allocation, patients with HCC were divided into three groups: mono-TACE group (75 patients), combo-TACE group (20 patients), and combo + RT group (14 patients). To compare the rate of HBV reactivation between the anti-HCC treatment and non-treatment groups, our analysis additionally included 62 HBsAg-negative patients as a control group subjected to regular monitoring of HBV markers as part of other observational studies (on the natural history of liver cirrhosis [16 patients] and HBsAg loss [46 patients]). Thus, the study consisted of observations in 109 patients with HCC and 62 control patients (Fig 1). The baseline characteristics of study participants are summarized in Table 1.

**Table 1:** Baseline characteristics of the study subjects.

	Treatment group (*n* = 109)	Control group (*n* = 62)
Age (years)	65.2 ± 12.2	58.4 ± 8.9
Sex (M:F, %)	85 (78.0):24 (22.0)	40 (64.5):22 (35.5)
ALT (IU/L)	31 (8–153)	26 (7–159)
Total bilirubin (mg/dl)	1.1 (0.2–3.7)	1.0 (0.2–10)
Child-Pugh classification (%)		
(A/B/C)	76 (69.7)/30 (27.5)/3 (2.8)	51 (82.3)/8 (12.9)/3 (4.8)
Anti-HBs positivity (%)	48 (44.0)/61 (56.0)	16 (25.8)/46 (74.2)
Anti-HBc positivity (%)	85 (78.0)/24 (22.0)	60 (96.8)/2 (3.2)

Treatment group = HBsAg-negative patients with HCC undergoing anti-cancer therapy (n = 109).

Control group = HBsAg-negative patients with cirrhosis (n = 16) and patients with HBsAg seroclearance (n = 46).

ALT, alanine aminotransferase.

### HBV reactivation and risk factors

**Fig 2:**
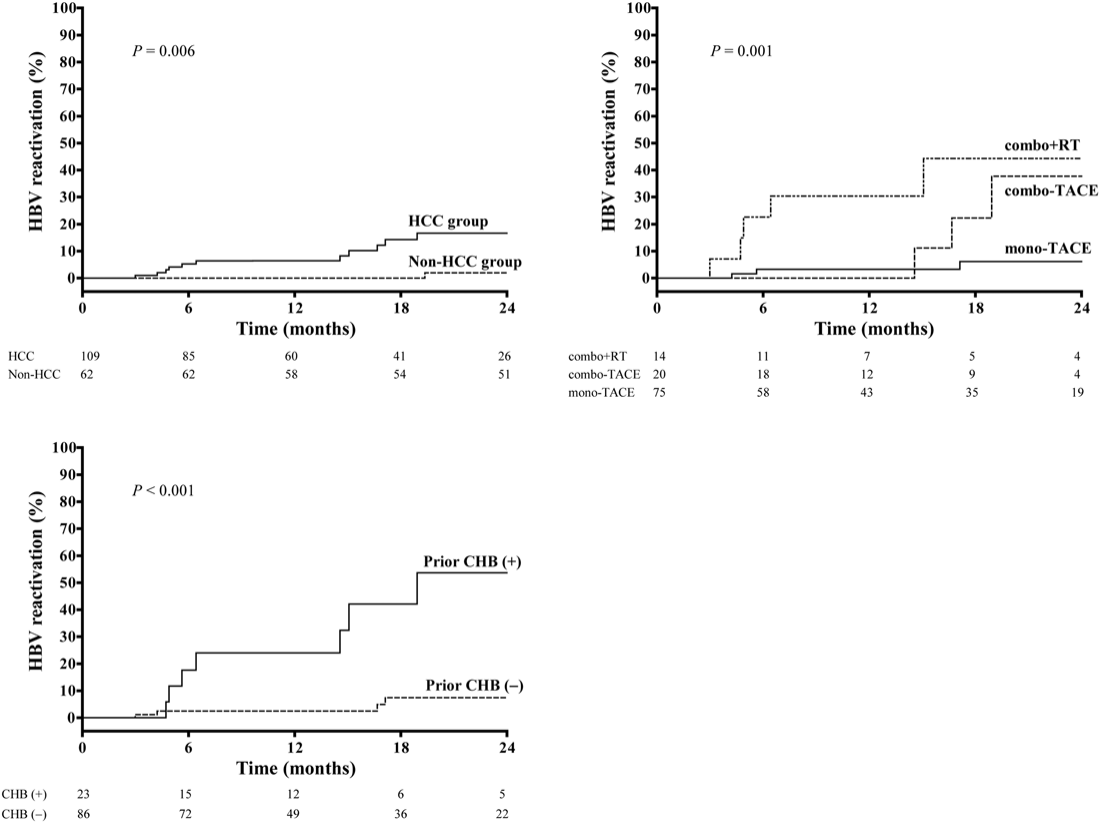
A, Kaplan-Meier curves of HBV reactivation for the HCC and control groups. Patients with HCC undergoing TACE had significantly more frequent episodes of HBV reactivation during follow-up than controls without TACE, with estimated 12- and 24-month rates of 7.7% vs. 0% and 15.9% vs. 2.0%, respectively (*P* = 0.006, log-rank test). B, Comparison of HBV reactivation between the HCC and control groups. The risk of HBV reactivation was highest with combo + RT, followed by combo-TACE and mono-TACE, with estimated 24-month rates of 44.3%, 37.8%, and 6.2%, respectively (*P* = 0.001, log-rank test). C, Comparison of HBV reactivation according to prior CHB status. Patients with prior CHB had a significantly higher incidence of HBV reactivation than those without, with estimated 24-month rates of 53.7% and 7.5%, respectively (*P* < 0.001, log-rank test).

In total, 13 (7.6%) patients, including 12 (11.0%) in the TACE group and 1 (1.6%) in the control group, eventually developed HBV reactivation during a median follow-up of 16.7 months (range: 0.43–24.0). Patients undergoing TACE experienced more frequent episodes of HBV reactivation than control patients, with an estimated probability of HBV reactivation of 7.7% and 0% at 12 months and 15.9% and 2.0% at 24 months in the TACE and control groups, respectively (*P* = 0.006; Fig 2A).

Analyses to determine predictors of HBV reactivation were performed for the entire population as well as HCC patients. For the entire study population, TACE was identified as the only independent predictor of HBV reactivation (hazard ratio [HR], 3.39, 95% confidence interval [CI]: 1.51–7.63; *P* = 0.003), while sex, age, liver enzymes, presence of anti-HBc or anti-HBs, and Child-Pugh class were not associated with reactivation (all *P* > 0.3). For 109 HCC patients undergoing TACE, 12 potential variables of interest were analyzed, as listed in Table 2. Of these, tumor morphology, type of treatment, and a prior history of chronic hepatitis B (CHB) were marginally or significantly associated with viral reactivation during therapy (Table 2). With multivariate analysis including these 3 factors, type of treatment (HR = 3.25; 95% CI, 1.60–6.58; *P* = 0.001) and presence of prior CHB (HR = 6.21; 95% CI, 1.84–20.83; *P* = 0.003) were finally identified as the two independent predictors of HBV reactivation.

**Table 2:** Risk factors for HBV reactivation in patients with HCC receiving transarterial therapy.

	No. of patients	HR	95% CI	*P* value
Sex				
Male	85	1.35	0.29–6.25	0.704
Female	24	1		
Age (years)				
>65	52	1.56	0.46–5.32	0.481
≤65	57	1		
Anti-HCV				
Ngative	83	1.59	0.34–7.34	0.556
Positive	26	1		
Anti-HBs				
Negative	48	1.62	0.49–5.35	0.428
Positive	61	1		
Anti-HBc				
Positive	86	1.71	0.38–7.69	0.487
Negative	23	1		
ALT (IU/L)				
≤40	72	1.15	0.31–4.37	0.831
>40	37	1		
Bilirubin (mg/dl)				
>1.2	73	1.85	0.56–6.07	0.311
≤1.2	36	1		
Tumor size (cm)				
5	65	1.76	0.54–5.80	0.352
5	44	1		
Tumor morphology				
Multi-nodular	57	3.39	0.89–12.91	0.073
Uni-nodular	52	1		
Treatment				
combo + RT	14	11.01	2.62–46.34	0.005*
combo-TACE	20	4.71	0.95–23.44	
mono-TACE	75	1		
Prior HBV disease				
Presence	85	5.95	1.82–19.60	0.003
Absence	24	1		
Child-Pugh class				
B/C	76	1.67	0.49–5.72	0.416
A	33	1		

HBV, hepatitis B virus; HCC, hepatocellular carcinoma; No, number; HR, hazard ratio; CI, confidence interval; ALT, alanine aminotransferase; HCV, hepatitis C virus; TACE, transarterial chemoembolization; RT, radiotherapy.

*P for trend.

### Type of treatment

When analyzed by type of treatment, the risk of HBV reactivation was highest in patients undergoing combo + RT, followed by combo-TACE and mono-TACE, with the adjusted HRs of 10.91 (95% CI, 2.49–47.80) for combo + RT and 4.63 (95% CI, 0.93–23.02) for combo-TACE, as compared with mono-TACE therapy (*P* for trend = 0.003). Kaplan-Meier plots for treatment option over time showed that the estimated 24-month probability of reactivation was 6.2%, 37.8%, and 44.3% for the mono-, combo-TACE, and combo + RT groups, respectively (*P* = 0.001; Fig 2B). These findings show a significant trend toward increasing incidence of HBV reactivation with increasing intensity of TACE.

### Prior history of hepatitis B

Among the 109 HBsAg-negative HCC patients, 23 (21.1%) had a prior history of medical record-confirmed CHB (prior CHB group), whereas the remaining 86 (78.9%) had no evidence of prior hepatitis B diseases (no prior CHB group). These 23 patients with prior CHB experienced more frequent episodes of HBV reactivation during TACE than those without, with estimated 12- and 24-month probabilities of reactivation of 24.0% and 53.7% for those with prior CHB and 2.5% and 7.5% for those without, respectively (*P* < 0.001; Fig 2C). The incidence of HBV reactivation between isolate anti-HBc-positive and HBsAb-positive/anti-HBc-positive patients among the HCC group was not different (11.6% [5/43] vs. 11.9% [5/42]; *P* = 0.968).

### Clinical outcomes of patients with HBV reactivation

**Fig 3:**
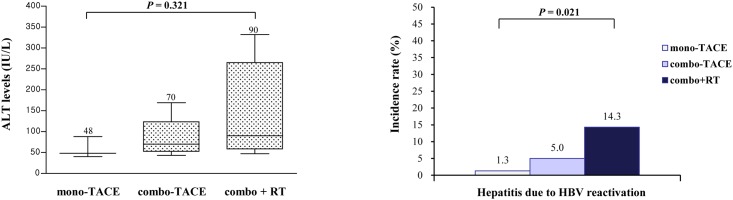
A and B, Clinical hepatitis associated with HBV reactivation according to treatment intensity. (A) ALT levels at HBV reactivation in reactivated patients (*P* = 0.321). (B) Hepatitis due to HBV reactivation according to treatment intensity among the entire patient group with HCC. The incidence rates of reactivation hepatitis were 1.3% (1/75) for mono-TACE, 5.0% (1/20) for combo-TACE, and 14.3% (2/14) for combo + RT (*P* for trend = 0.021).

The clinical features of the 13 patients with reactivation are shown in Table 3. Overall, 4 (30.8%) patients in the TACE group and none in the control group developed clinical hepatitis due to HBV reactivation during follow-up. For HCC patients with HBV reactivation, the median times elapsed to HBV reactivation and reactivation hepatitis were 6.77 months (range: 3.0–18.93) from initiation of TACE and 84.5 (range: 41–443) days from the onset of viral reactivation, respectively. The median levels of HBV DNA and ALT for the 12 HCC patients with HBV reactivation were 5,174 copies/ml (range: 216–116,058) and 74 IU/L (range: 40–332) at the time of HBV reactivation. The levels of ALT and incidence of hepatitis due to HBV reactivation tended to correlate positively with the type of TACE, with a higher risk of hepatic events with increasing TACE intensity (Fig 3A and 3B).

**Table 3:** Clinical features of 13 HBsAg-negative patients who developed HBV reactivation during the follow-up.

		Baseline			Treatment		At the time of HBV reactivation					Management			
Pt	Sex/Age	HBsAg/Abstatus	Anti-HBc state	Past HBV infection	Type of treatment	No of TACE cycles to reactivation	Time to HBV reactivation (months)	HBsAg	Peak ALT (IU/L)	HBV DNA (copies/ml)	Development of decompen-sation	Antiviral treatment	Further treatment session	Follow-up viral markers (time after reactivation)	Outcome
1	M/58	-/-	+	No	Mono	3	17.13	-	40	9,557	No	No	Not affected	Not checked	Died of SDH (18 mo)
2	M/76	-/+	+	Yes	Mono	2	5.63	+	48	66,858	No	No	Not affected	Spontaneous HBsAg loss7 mo later	Alive (26 mo)
3	M/73	-/-	+	Yes	Combo+RT	1	6.43	-	70	2,189	No	No	Not affected	Spontaneous decreasein HBV DNA levels	Died of PD (9 mo)
4	M/73	-/+	+	No	Combo	9	16.66	-	62	216	No	No	Not affected	HBV DNA fluctuatingfrom UD to 10^3^ copies/ml	FU loss (45 mo)
5	M/67	-/-	+	Yes	Combo+RT	5	15.06	+	332	30,016	Yes (ascites)	Lamivudine	Delayed	HBV DNA UD at 2 mo	Died of PD (19 mo)
6	M/54	-/-	+	Yes	Combo	9	14.57	+	169	8,160	No	Entecavir	Not affected	HBV DNA UD at 2 mo	Alive (33 mo)
7	M/69	-/-	+	Yes	Combo	5	18.93	+	43	1,540	No	Entecavir	Not affected	HBsAg loss 26 mo later	Alive (51 mo)
8	M/68	-/+	+	No	Mono	2	4.23	+	88	116,058	No	Lamivudine	Not affected	HBsAg loss 2 mo later	Died of Vx bleed (7 mo)
9	M/61	-/+	+	No	Combo+RT	3	3.00	-	90	379	No	No	Not affected	HBV DNA fluctuatingbelow 10^2–3^ copies/ml	Alive (7 mo)
10	F/61	-/-	+	Yes	Combo+RT	3	4.90	+	47	14,219	No	Entecavir	Not affected	HBsAg loss 7 mo later	Died of PD (9 mo)
11	M/77	-/+	+	Yes	Combo+RT	4	4.73	-	198	1,293	No	Lamivudine	Not affected	HBV DNA UD at 1 mo	Died of PD (19 mo)
12	F/48	-/+	-	No	Combo	6	8.46	-	78	750	No	No	Not affected	Spontaneous decreasein HBV DNA levels	Alive (60 mo)
13	F/53	-/-	+	Yes	No (control group)	NA	19.37	-	41	677	No	No	NA	HBV DNA fluctuatingbelow 10^3^ copies/ml	Alive (24 mo)

HBsAg, hepatitis B surface antigen; HBV, hepatitis B virus; Pt, patient; M, male; F, female; TACE, transarterial chemoembolization; Mono, mono-drug TACE; Combo, combination-drug TACE; RT, radiotherapy; mo, months; UD, undetectable; No, number; Tx, treatment; ALT, alanine aminotransferase; SDH, subdural hemorrhage; PD, progressive disease; FU, follow-up.

Under the stringent national insurance policy, antiviral therapy was administered as early as possible for any patients who met the criteria (HBV DNA > 10^4–5^ copies/ml with increased ALT levels > 2 × ULN). As a result, antiviral treatment was commenced for six (50.0%) of the 12 HCC patients with reactivation, including 3 meeting these criteria and 3 who agreed to have treatment without reimbursement, all of whom achieved early viral suppression. Six (50.0%) of the 12 patients had HBsAg sero-reversion at the time of reactivation, but 4 (4/6, 66.7%) eventually developed HBsAg seroclearance during the extended follow-up. However, one (8.3%) patient with HBV reactivation developed mild ascites, on which the diagnosis of hepatic decompensation was made. This patient suffered from reactivation after combo + RT therapy and further scheduled TACE sessions were delayed in the patient. Lamivudine was immediately initiated, and liver function improved, but the patient ultimately died due to tumor progression. The remaining 6 antiviral-untreated patients with HBV reactivation eventually experienced spontaneous HBsAg loss (one patient) or low HBV DNA levels (5 patients) fluctuating below 10^4^ copies/ml without significant ALT elevations throughout follow-up without antiviral therapy.

## Discussion

Until recently, HBV reactivation among HBsAg-negative patients was almost entirely neglected as a research topic except in the setting of hematologic diseases. The present study, however, demonstrates that HBV reactivation occurs at a rate of 11.0% in HBsAg-negative patients undergoing TACE-based therapy for HCC. This non-negligible incidence indicates that patients with HCC should be considered a high-risk group for HBV reactivation, although there is no such specification in the current practice guidelines [10–12]. Reactivation of HBV is caused by anti-HCC treatment, as viral reactivation in the treatment group was significantly more frequent than in the control group. It is noteworthy that while most patients with reactivation responded well to antiviral therapy, one patient developed hepatic decompensation leading to interruption of the TACE schedule. The overall findings raise concerns about the potential for HBV reactivation and the need for appropriate management during TACE in HBsAg-negative as well as HBsAg-positive patients.

It is presumed that TACE, unlike systemic chemotherapy, does not directly cause reactivation, as it has little effect on host immunity. However, TACE does have systemic effects via peritumoral circulation, as evidenced by a series of reactivation cases during TACE [2]. Large HCCs are likely to contain a more complex vasculature including an arterio-venous shunt around the tumor, and thus, the chemotherapeutic agent primarily infused into the tumor is easily released into systemic circulation, sometimes causing immune suppression sufficient for HBV reactivation. This has been supported by the previous observations, indicating the higher tendency for reactivation in patients with advanced HCC [15]. On the other hand, liver-directed chemotherapy can result in direct liver damage and altered regulation of hepatic inflammation and immunity, as the liver is a major immune organ [16]. The additive effect of viral reactivation in addition to TACE-induced liver damage can intensify necroinflammatory liver injury, adversely affecting patient outcome.

For chronic HBV carriers, reactivation may occur either spontaneously or after immuno-chemotherapy [3]. Based on our results, it appears obvious that the reactivation phenomenon is not spontaneous, but rather is caused by anti-HCC treatment, as almost all reactivation cases occurred in treated patients, while spontaneous reactivation was quite rare (1/62, 1.6%) in untreated patients. Despite the lower seropositivity of anti-HBc, a marker for prior exposure to HBV, the treatment group showed a higher rate of reactivation than the control group. Given the current lack of comparative data between spontaneous and chemotherapy-induced reactivation of HBV, this analysis offers valuable evidence confirming that HBV reactivation is induced by anti-cancer therapy.

Occult HBV infection has been suggested to have a role in chemotherapy-induced HBV reactivation in HBsAg-negative patients [5]. One may argue that viral reactivation observed in our patients merely represents the typical pattern of fluctuating HBV DNA at low levels, occasionally falling or rising between undetectable and detectable ranges in occult HBV carriers. However, reactivated cases in the current study demonstrated both a greater increase in viral load and a greater rate of clinical hepatitis, which are not typically seen in occult HBV carriers without treatment. In our analysis, a past history of CHB is more helpful in risk assessment of reactivation for HBsAg-negative HCC patients. Thus, it is necessary to carefully scrutinize a prior medical history of CHB in the management of HBsAg-negative patients who are to undergo therapy for HCC. Anti-HBc screening is also relevant in HBsAg-negative patients on chemotherapy, as it helps to identify patients with past infection of HBV who will be potentially predisposed to reactivation under immunosuppression due to HBV persistence after resolution. Indeed, all but one (12/13, 92.3%) of our reactivation cases were positive for anti-HBc. This indicates that anti-HBc testing may serve as a negative predictive indicator for reactivation during therapy of HBsAg-negative HCC.

As with HBsAg-positive patients [15], intensive chemo-radiotherapy (combo + RT) was more commonly associated with an increased incidence and severity of reactivated diseases among HBsAg-negative patients, while those undergoing non-intense mono-TACE rarely experienced reactivation. This implies that in either HBsAg-positive or -negative cases, viral reactivation is essentially the same phenomenon proportionate to the level of immunosuppression, but its frequency varies according to the level of baseline viremia. Based on our results, type of therapy and past history of CHB represent relevant factors that should be considered when establishing preventive measures against HBV reactivation in this particular population.

The management guidelines issued by the European Association for the Study of the Liver recommend pretreatment HBsAg and anti-HBc screening for all candidates for chemotherapy [10]. It also recommends that HBV DNA should be tested at baseline and during follow-up for HBsAg-negative/anti-HBc-positive patients, and antiviral therapy be initiated upon evidence of detectable HBV DNA [10]. However, the recommendations of the American Association for the Study of Liver Diseases lack these specifications [11]. Importantly, our findings argue for the use of anti-HBc screening in addition to HBsAg for all patients who are to receive chemotherapy as well as close examination for prior CHB infection.

Although prophylactic antiviral therapy has recently been shown to prevent HBV reactivation in patients with HBsAg-negative/anti-HBc-positive lymphoma [17], the role of antiviral prophylaxis is unknown in HBsAg-negative patients with HCC. In our series, reactivation occurred commonly after intense TACE, but was rare after conventional TACE-monotherapy. Most instances of reactivation were quite responsive to antiviral therapy. These findings suggest that conventional, modest-intensity TACE is associated with a low risk of reactivation and thus, the routine use of prophylaxis may not provide a competitive advantage over deferred therapy upon evidence of HBV reactivation in these patients. By contrast, patients with a prior history of CHB undergoing intense TACE may require an appropriate management against HBV reactivation, as reactivated disease is potentially fatal [4]. Notably, 6 patients with reactivation did not suffer any adverse outcomes or had spontaneous control of viremia without antiviral therapy, as opposed to patients treated with antivirals. This highlights the complexity of the host-viral interactions at play during HBV reactivation and underscores the need for prospective studies to address the many issues surrounding the management of HBV reactivation in HBsAg-negative patients.

This study had some limitations. It was a non-randomized, single-center study. The clinical features of reactivation can vary among institutions employing different treatment options. The role of occult HBV infection was not evaluated in this study, due to the lack of specific analysis to detect occult infection in patients’ serum or liver tissues. Thus, potential inclusion or exclusion of true occult carriers in this analysis might affect the results. Nevertheless, the consecutive nature of patient enrollment and regular monitoring of HBV markers over an acceptable follow-up period would increase the reliability of the study results.

In conclusion, the present study highlights that TACE can reactivate HBV replication in HBsAg-negative patients, with a dose-risk relationship between reactivated disease and increasing intensity of treatment. Considering the relatively low incidence of HBV reactivation, low levels of HBV DNA at reactivation, and good efficacy of antiviral therapy, anti-HBV therapy can be deferred until HBV DNA becomes detectable in HBsAg-negative patients undergoing conventional TACE. However, as a high-risk group for reactivation, patients with a prior history of CHB undergoing high-intensity TACE should be carefully monitored for HBV markers during TACE to facilitate early initiation of antiviral therapy, with an alternative approach of antiviral prophylaxis against HBV reactivation.
